# Comparing Genetic and Socioenvironmental Contributions to Ethnic Differences in C-Reactive Protein

**DOI:** 10.3389/fgene.2021.738485

**Published:** 2021-10-18

**Authors:** Shashwat Deepali Nagar, Andrew B. Conley, Shivam Sharma, Lavanya Rishishwar, I. King Jordan, Leonardo Mariño-Ramírez

**Affiliations:** ^1^ School of Biological Sciences, Georgia Institute of Technology, Atlanta, GA, United States; ^2^ IHRC-Georgia Tech Applied Bioinformatics Laboratory, Atlanta, GA, United States; ^3^ National Institute on Minority Health and Health Disparities, National Institutes of Health, Bethesda, MD, United States; ^4^ PanAmerican Bioinformatics Institute, Cali, Colombia

**Keywords:** C-reacitve protein, inflammation, race and ethnicity (minority issues), health disparities, genetic ancestry, socioeconomic stages

## Abstract

C-reactive protein (CRP) is a routinely measured blood biomarker for inflammation. Elevated levels of circulating CRP are associated with response to infection, risk for a number of complex common diseases, and psychosocial stress. The objective of this study was to compare the contributions of genetic ancestry, socioenvironmental factors, and inflammation-related health conditions to ethnic differences in C-reactive protein levels. We used multivariable regression to compare CRP blood serum levels between Black and White ethnic groups from the United Kingdom Biobank (UKBB) prospective cohort study. CRP serum levels are significantly associated with ethnicity in an age and sex adjusted model. Study participants who identify as Black have higher average CRP than those who identify as White, CRP increases with age, and females have higher average CRP than males. Ethnicity and sex show a significant interaction effect on CRP. Black females have higher average CRP levels than White females, whereas White males have higher average CRP than Black males. Significant associations between CRP, ethnicity, and genetic ancestry are almost completely attenuated in a fully adjusted model that includes socioenvironmental factors and inflammation-related health conditions. BMI, smoking, and socioeconomic deprivation all have high relative effects on CRP. These results indicate that socioenvironmental factors contribute more to CRP ethnic differences than genetics. Differences in CRP are associated with ethnic disparities for a number of chronic diseases, including type 2 diabetes, essential hypertension, sarcoidosis, and lupus erythematosus. Our results indicate that ethnic differences in CRP are linked to both socioenvironmental factors and numerous ethnic health disparities.

## Introduction

C-reactive protein (CRP) is synthesized by hepatocytes and secreted to the bloodstream in response to inflammation. CRP is employed as a serum biomarker for both acute and chronic inflammation, with important implications for immune response and overall health (Pepys and Hirschfield 2003; Black et al., 2004). Elevated levels of CRP have been shown to be associated with an increased risk of diabetes (Dehghan et al., 2007), cardiovascular disease (Danesh et al., 2004), psychological stress (Wium-Andersen et al., 2013), and all-cause mortality (Zacho et al., 2010).

CRP blood serum levels vary across ethnic groups (Nazmi and Victora 2007), with a number of studies showing that Black patients have higher average levels of circulating CRP than White patients (Wong et al., 2001; Abramson et al., 2002; Ford 2002; Danner et al., 2003; Khera et al., 2005; Matthews et al., 2005; Alley et al., 2006; Lakoski et al., 2006). Ethnic differences of this kind are likely to have multifactorial causes, including contributions from genetic, socioeconomic, and environmental factors. Given the fact that ethnicity co-varies with all of these classes of risk factors, it is difficult to tease apart the genetic and socioenvironmental contributions to ethnic health disparities. This is further complicated by the fact that socially defined ethnicity is an imprecise proxy for genetic diversity.

We use genetic ancestry inference as a means to disambiguate genetic and socioenvironmental effects on ethnic health disparities. Genetic ancestry refers to patterns of genetic diversity that are linked to the geographical origins of human populations (Mathieson and Scally 2020). Individuals who share common ancestors have genetic similarities, and distinct ancestry groups show correlated allele frequency differences (Bergstrom et al., 2020; Genomes Project et al., 2015). Genetic ancestry can be defined objectively, using comparative genomic analysis, without relying on socially defined ethnic groups (Yudell et al., 2016). Patterns of genetic ancestry can be compared to self-identified ethnicity to understand the extent to which they overlap and how they may differ (Tang et al., 2005; Banda et al., 2015; Fang et al., 2019). Modelling of health outcomes with genetic ancestry and socioenvironmental factors as independent (predictor) variables can be used to assess how each contribute to health disparities and how they may interact (Choudhry et al., 2007; Nagar et al., 2021).

The objective of this study was to characterize the effects of genetic ancestry, socioenvironmental factors, and health conditions on ethnic disparities in CRP serum levels. Participants from the United Kingdom Biobank (UKBB) prospective cohort study who self-identified as belonging to Black or White ethnic groups were characterized with respect to CRP levels, genome-wide genotypes, and a variety of socioenvironmental factors and health conditions that show prevalence disparities across groups. Multivariable regression and relative importance analysis were used in an effort to decompose genetic and socioenvironmental contributions to CRP ethnic disparities. Age and sex were considered as covariates in all models given their known associations with CRP serum levels. Given that genetic and socioenvironmental contributions to health outcomes can co-vary across ethnic groups, we hypothesized that the inclusion of socioenvironmental covariates could attenuate the association between ethnicity, genetic ancestry, and CRP levels.

## Materials and Methods

### Study Cohort

Study participants and data were taken from the United Kingdom Biobank (UKBB), a prospective cohort study on the effects of demography, environment, and genetics on health and disease (Bycroft et al., 2018). The UKBB database contains phenotypic, clinical, and genetic information on more than 500,000 participants between the ages of 40 and 70, enrolled from 2006 to 2010. Ethics approval for the UKBB was obtained from the North West Multi-centre Research Ethics Committee (MREC) for the United Kingdom, the Patient Information Advisory Group (PIAG) for England and Wales, and the Community Health Index Advisory Group (CHIAG) for Scotland^1^. UKBB participants self-identified as belonging to a single ethnic group upon enrollment^2^, and we included participants who identified as Black or White for this study. It should be noted that the UKBB ethnic group labels used here correspond directly to racial group labels from the United States. This study was conducted under the United Kingdom Biobank project #65206 to LMR and IKJ.

### Participant Data

UKBB participants completed questionnaires, nurse-led interviews, and medical assessments upon enrollment and provided access to their electronic health records. We accessed participant information on age (Field 21003: Age when attended assessment center), BMI (Field 21001: Body mass index (BMI), ethnicity (Field 21000: Ethnic background), ICD-10 disease diagnosis codes (Fields 41270: Diagnoses–ICD10), insomnia (Field 1200: Sleeplessness/insomnia), recruitment year (Field 53: Date of attending assessment centre), sex (Field 31: Sex), smoking status (Field 20116: Smoking status), and Townsend deprivation index (Field 189: Townsend deprivation index at recruitment) from the UKBB data portal. If multiple instances of participant data were available from a follow-up visit, only data collected during the initial assessment visit (2006–2010) were used for analysis.

The Townsend deprivation index is a widely used measure of socioeconomic deprivation known to be associated with poor health outcomes (Foster et al., 2018). It combines four variables–unemployment, non-car ownership, non-home ownership, and household overcrowding–to generate a numerical score (Townsend et al., 1988), which ranges from −6.26 to 11.0 in the UKBB study cohort. Negative values indicate less socioeconomic deprivation, and relative affluence, whereas higher scores indicate greater socioeconomic deprivation.

UKBB participants provided whole blood samples for characterization of protein biomarkers and DNA as previously described (Elliott et al., 2008). C-reactive protein (CRP) blood serum levels were measured as mg/L units using the immuno-turbidimetric method with the Beckman Coulter AU5800 clinical chemistry analyzer^3^. This procedure corresponds to the high-sensitivity (hs) CRP test. DNA was extracted from 850 μl buffy coat blood aliquots^4^, and participant genome-wide genotypes were characterized using the UKBB Axiom Array or United Kingdom BiLEVE Array (Welsh et al., 2017).

### Disease Case/Control Cohorts

Disease (or health condition) diagnoses for study participants were taken from UKBB ICD-10 diagnosis codes, which were then converted into disease-specific phenotype codes (phecodes) using the scheme developed by the PheWAS consortium (Carroll et al., 2014). Phecodes have been manually curated and validated by disease experts, and they are widely used for the analysis of electronic health record data (Wu et al., 2019). The phecode scheme provides ICD-10 code inclusion and exclusion criteria for each individual disease in order to define disease-specific case/control cohorts that can be confidently compared. For example, when studying participants with type 2 diabetes, participants with type 1 diabetes are removed from the control cohort to avoid any overlapping genetic or environmental signals that might be common to both. This approach improves power for the detection of disease-specific association signals when modelling case/control status. Phecode case/control cohorts were curated for a total of 1,537 diseases or health-related conditions.

### Genetic Ancestry Inference

UKBB participant genome-wide genotypes were merged and harmonized with whole genome sequence data from global reference populations characterized as part of the 1,000 Genomes Project (1KGP) and the Human Genome Diversity Project (HGDP) (Bergstrom, et al., 2020; Genomes Project, et al., 2015). Global reference populations were grouped into six regional ancestry groups based on their genetic and geographic affinity, including African (sub-Saharan) and European reference population groups (Supplementary Table 1).

UKBB, 1KGP, and HGDP genomic variant data were merged to include variants present in all three data sets. Minor allele frequency >1% and variant sample missingness <5% filters were used for merging, with variant strand flips and identifier inconsistencies corrected as needed. The merged genome variant data set was pruned for linkage disequilibrium using the program PLINK v2 (Chang et al., 2015).

Principal component analysis (PCA) of the harmonized UKBB, 1KGP, and HGDP genome variant dataset was performed using the FastPCA program implemented in PLINK v2 (Galinsky et al., 2016). Data for the first ten PCs were used to infer UKBB participant genetic ancestry fractions for African, European, and other regional ancestry groups. Our PCA-based genetic ancestry inference approach compares PCA data from UKBB participants to PCA data from reference population individuals using non-negative least squares to assign genetic ancestry fractions for regional ancestry groups as previously described (Conley et al., 2017; Jordan et al., 2019). Participants showing >5% non-African or non-European ancestry fractions were excluded from the study cohort.

### Statistical Modeling

All statistical analyses were performed using the R statistical language v3.6.1 (Team R. C, 2013). Forest plots were generated using the forestmodel R package (Kennedy 2020). Other plots were generated using the ggplot R package (Wickham 2009).

CRP blood serum levels are measured in mg/L units by the UKBB. Log transformed CRP blood serum levels were modeled as a continuous outcome using linear regression, and clinically elevated CRP (>3 mg/L) was modeled as a binary outcome using logistic regression using the “glm” function in R. Independent (predictor) variables for the base models included ethnicity, age, and sex. Ethnicity was modeled as a binary variable (Black or White), age was mean centered and modeled as a continuous variable, and sex was modeled as a binary variable (female or male). Independent (predictor) variables for the fully adjusted models included BMI, insomnia, major depressive disorder, PC1, and PC2, recruitment year, smoking status, and the Townsend deprivation index. BMI, PC1, and PC2, and the Townsend index were modeled as continuous variables, and insomnia and smoking status were modeled as categorical variables, and major depressive disorder was modeled as a binary variable. Relative importance analysis was used to assess the relative contributions of the different covariates in the fully adjusted model, while accounting for multicollinearity. The R package “relaimpo” was used for analysis.

The odds of prevalence of specific diseases or health conditions were modeled as the dependent (outcome) variable with multivariable logistic regression computed using the “glm” function in R. Independent (predictor) variables for disease models included ethnicity, mean centered age, sex, and log transformed CRP levels.

Model specifications and detailed results for all statistical models are provided in the Supplementary Material. For each model, we provide the regression equation and model coefficients, along with effect size estimates, standard errors, z-values, and *p*-values for each model coefficient.

## Results

### C-Reactive Protein, Ethnicity, Age, and Sex

**FIGURE 1:**
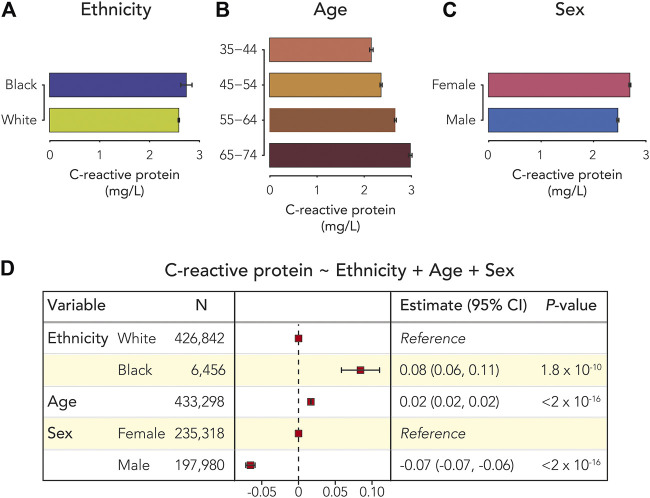
C-reactive protein (CRP), ethnicity, age, and sex. Average CRP serum levels (±95% CI) are shown for **(A)** Black and White participants **(B)** age ranges, and **(C)** female and male participants. **(D)** Forest plot showing the results of the multivariable linear regression model of participant CRP serum levels. Effect sizes, 95% CIs, and *p*-values are shown for ethnicity, age, and sex.

The study cohort is made up of 433,298 United Kingdom Biobank participants who self-identify as Black (*n* = 6,456) or White (*n* = 426,842) (Table 1). Males make up 45.7% of the cohort compared to 54.3% females, and the mean age of cohort participants is 57. C-reactive protein (CRP) blood serum levels vary by ethnicity, age, and sex. Black participants show a mean CRP level of 2.75 mg/L, and White participants show mean CRP level of 2.59 mg/L (t = 2.72, *p* = 0.01; Table 1 and Figure 1A). 25% of Black participants have clinically elevated CRP compared to 22.6% of White participants (Table 1). Participant CRP levels increase with increasing age (Figure 1B), and females show higher mean CRP levels than males (t = 17.22, *p* < 2.2 × 10^−16^; Figure 1C). When CRP levels are modeled by ethnicity, age, and sex, Black ethnicity and age show significant positive associations with CRP, whereas male sex shows a significant negative association with CRP (ethnicity: *β* = 0.08, *p* = ∼0, age: *β* = 0.02, *p* = ∼0, sex: *β* = −0.06, *p* = ∼0; Figure 1D and Supplementary Table 2). These results are robust to a sensitivity analysis conducted by removing individuals with high levels of CRP (≥10 mg/L), indicative of acute infections (Supplementary Table 3). Similarly, when clinically elevated CRP (>3 mg/L) is modeled as a binary outcome, Black ethnicity and age show significant positive associations, and male sex shows a significant negative association (ethnicity: *β* = 0.04, *p* = ∼0, age: *β* = 0.003, *p* = ∼0, sex: *β* = −0.04, *p* = ∼0; Supplementary Table 4).

**TABLE 1:** Characteristics of the United Kingdom Biobank participant cohort.

**Characteristic**	**Full cohort**	**Black**	**White**
N	433,298	6,456	426,842
Mean age, years (sd)	56.71 (8.05)	52.08 (8.08)	56.78 (8.02)
Sex—no. (%)			
Female	235,318 (54.31)	3,721 (57.64)	231,597 (54.26)
Male	197,980 (45.69)	2,735 (42.36)	195,245 (45.74)
Mean C-reactive protein, mg/L (sd)	2.60 (4.37)	2.75 (4.5)	2.59 (4.37)
Clinically elevated C-reactive protein, % > 3 mg/L	22.62	25.0	22.58
Mean European ancestry % (sd)	98.45 (10.77)	11.71 (10.52)	99.76 (0.77)
Mean African ancestry % (sd)	1.33 (10.72)	87.81 (10.50)	0.03 (0.46)
Mean Townsend index (sd)	−1.42 (3.03)	2.63 (3.42)	-1.48 (2.98)

**FIGURE 2:**
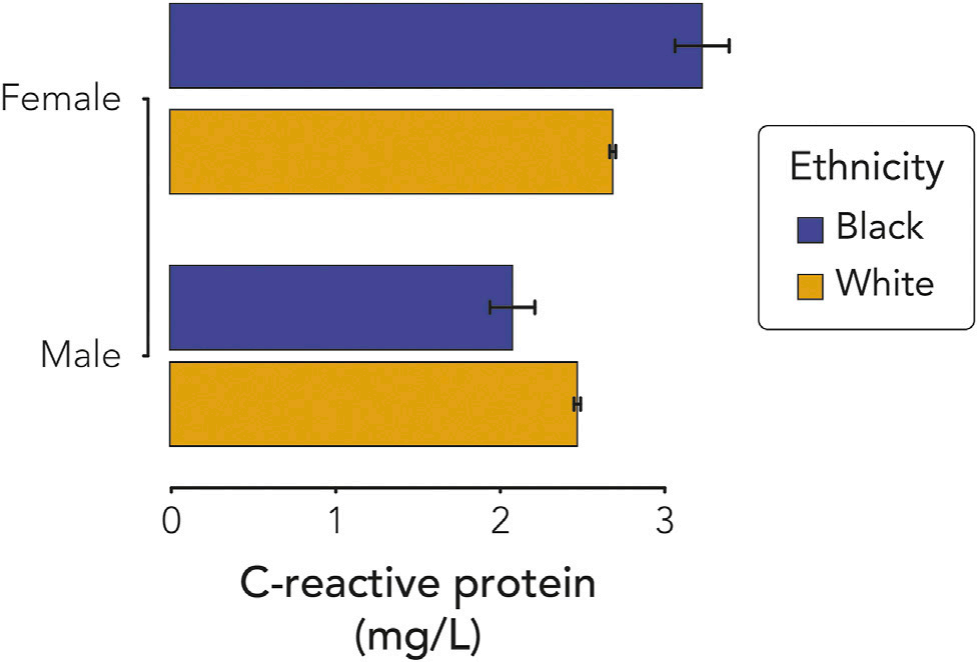
C-reactive protein (CRP) interaction effect of sex and ethnicity. Average CRP serum levels (±95% CI) are shown for Black and White, female, and male participants.

Inclusion of interaction terms in the CRP linear regression model revealed a significant interaction between ethnicity and sex (Supplementary Table 5). A likelihood ratio test showed a significantly better fit for a model with the ethnicity-sex interaction term compared to the model with no interaction term, providing additional support for the interaction (Supplementary Table 6). The observed ethnicity-sex interaction results from higher CRP for Black female participants compared to White female participants and lower CRP for Black male participants compared to White male participants (Figure 2).

### Ethnicity and Genetic Ancestry

Black and White participants differ with respect to mean levels of African and European genetic ancestry (Table 1). Black participants show averages of 87.8% African ancestry and 11.7% European ancestry compared to averages of 99.8% European and 0.03% African ancestry for White participants (African ancestry: t = 671.91, *p* < 2.2 × 10^−16^; European ancestry: t = 672.38, *p* < 2.2 × 10^−16^).

**FIGURE 3:**
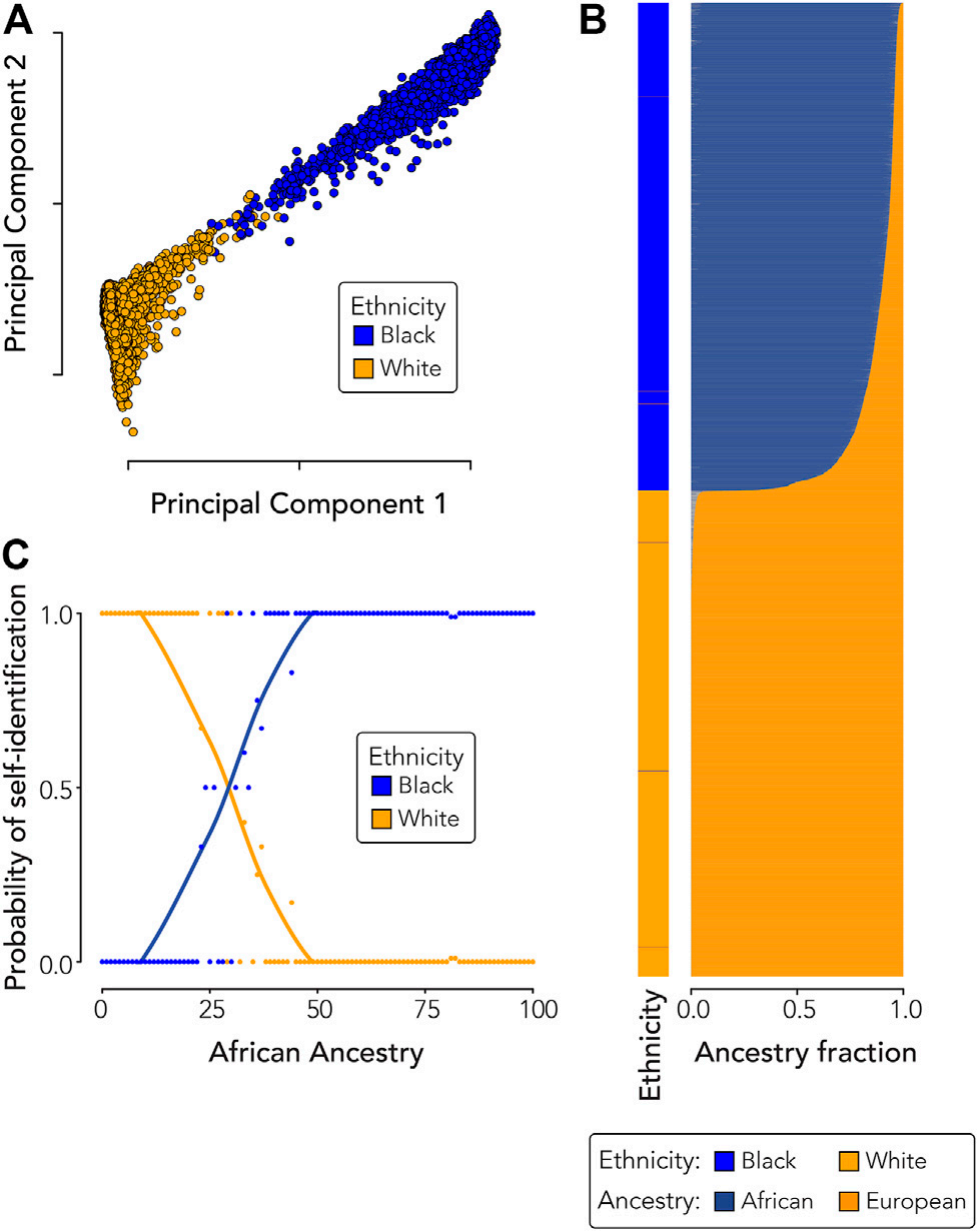
Ethnicity and genetic ancestry. **(A)** Principal components analysis showing the relationship between participant ethnicity and genetic diversity. **(B)** Participant ethnicity (left) compared to participant genetic ancestry fractions (right). **(C)** Probability of participant ethnic self-identity (*y*-axis) compared to African genetic ancestry (*x*-axis). Dots are color-coded by participant ethnicity and each dot shows the probability of self-identification as Black or White across one hundred bins of African ancestry. Black and White self-identification probability trend lines were fit using loess regression.

The relationship between ethnicity and genetic diversity for Black and White participants can be visualized via principal components analysis of genome-wide genotype data (Figure 3A). Principal components one and two separate participants by ethnicity along a continuum of genetic diversity, whereas principal component two alone shows more within ethnic group differences among participants. Black participants show a range of admixture between African and European genetic ancestry fractions, whereas White participants show almost entirely European ancestry (Figure 3B). The probability of participant self-identification as Black or White shifts in the range of 23–44% African ancestry (Figure 3C). Participants are more likely to identify as Black, and less likely to identify as White, if they have ≥29% African ancestry.

### Genetic and Socioenvironmental Effects on C-Reactive Protein Ethnic Disparities

Multivariable regression and relative importance analysis were used to model the effects of ethnicity, genetic ancestry, socioenvironmental factors, and health conditions that disproportionately affect different ethnic groups on CRP serum levels. Genetic ancestry was modeled using the first two principal components, which show the greatest separation between African and European ancestry components. Socioeconomic deprivation was modeled using the Townsend index, BMI, insomnia, and major depressive disorder (MDD) were included as disparate health conditions, and recruitment year and smoking were included as environmental exposures. The effect of recruitment year was considered to account for possible psychosocial stress associated with the financial crisis of 2007–2008. The effects of all covariates on CRP were considered separately using age and sex adjusted models and together with a fully adjusted model (Table 2 and Supplementary Table 7).

**TABLE 2:** C-reactive protein multivariable linear regression models. Covariates are shown along with the category labels for categorical variables. The effect of each covariate on CRP serum levels was considered separately with age and sex adjusted models and together in the fully adjusted model. Effect point estimates are shown for each covariate along with standard errors and *p*-values in parentheses. The relative importance rank of each covariate in the fully adjusted model is shown.

Covariate	Category	Age and sex adjusted	Fully adjusted model	Relative importance rank
Ethnicity	White	Reference	Reference	11
	Black	0.30 (0.05, 2.67 × 10^−08^)	0.43 (0.41, 2.9 × 10^−1^)	
Age	—	—	0.03 (0, 4.83 × 10^−246^)	2
Sex	Female	Reference	Reference	5
	Male	—	−0.46 (0.01, 4.44 × 10^−254^)	
BMI	—	0.23 (0.00, ∼0)	0.22 (0.0, ∼0)	1
Insomnia	Never/rarely	Reference	Reference	6
	Prefer not to answer	0.36 (0.24, 1.33 × 10^−1^)	0.1 (0.25, 6.91 × 10^−1^)	
	Sometimes	0.13 (0.02, 3.80 × 10^−14^)	0.05 (0.02, 1.56 × 10^−3^)	
	Usually	0.44 (0.02, 2.43 × 10^−120^)	0.16 (0.02, 8.22 × 10^−19^)	
MDD	—	1.09 (0.19, 1.37 × 10^−8^)	0.68 (0.19, 2.80 × 10^−4^)	8
Recruitment Year	—	−0.06 (0.01, 1.23 × 10^−17^)	−0.07 (0.01, 9.28 × 10^−19^)	7
Smoking	Never	Reference	Reference	3
	Current	1.01 (0.02, ∼0)	0.84 (0.02, 1.97 × 10^−284^)	
	Prefer not to answer	0.69 (0.11, 4.47 × 10^−10^)	0.24 (0.12, 4.26 × 10^−2^)	
	Previous	0.30 (0.01, 8.46 × 10^−95^)	0.05 (0.01, 4.35 × 10^−4^)	
PC1	—	0.001 (0.0, 1.19 × 10^−7^)	0 (0, 4.38 × 10^−3^)	9
PC2	—	0.004 (0.00, 1.96 × 10^−8^)	0.01 (0, 6.24 × 10^−2^)	10
Townsend Index	—	0.10 (0.00, ∼0)	0.04 (0, 2.07 × 10^−78^)	4

The significant positive association between Black ethnicity and CRP levels is attenuated in the fully adjusted model, and ethnicity shows the lowest relative importance among the 11 covariates included in the model. The effects of genetic ancestry are similarly attenuated in the fully adjusted model and have the next lowest relative importance values (PC1 = 9, PC2 = 10). BMI has the highest relative effect on CRP in the fully adjusted model followed by age, smoking, socioeconomic deprivation measured by the Townsend index, and sex. Insomnia, recruitment year, and MDD all show significant but marginally important effects on CRP. Considered together, these results are consistent with the hypothesis that ethnic differences in socioenvironmental exposures and health outcomes explain the association between ethnicity, genetic ancestry, and CRP levels.

### C-Reactive Protein and Ethnic Health Disparities

**FIGURE 4:**
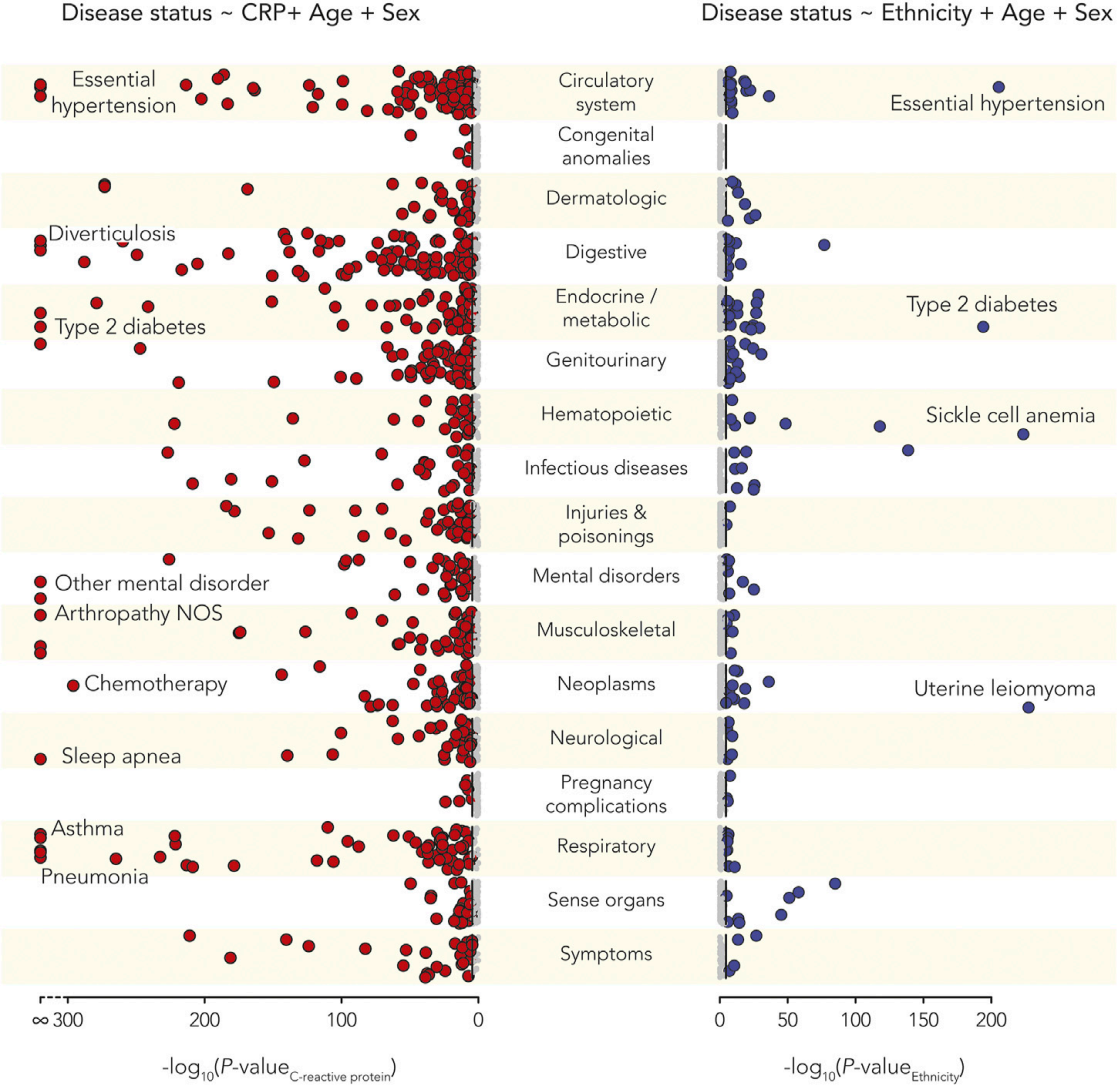
C-reactive protein (CRP) and ethnic health disparities. Manhattan plots showing the statistical significance levels (-log_10_ *p*-values) for associations of disease and CRP (left, red circles) and associations of disease and ethnicity (right, blue circles). Diseases are categorized as shown using the phecode scheme. Bonferroni corrected *p*-value thresholds are shown with black lines. Bonferroni corrected significant associations are indicated with larger, dark circles and select disease examples are annotated as shown.

**FIGURE 5:**
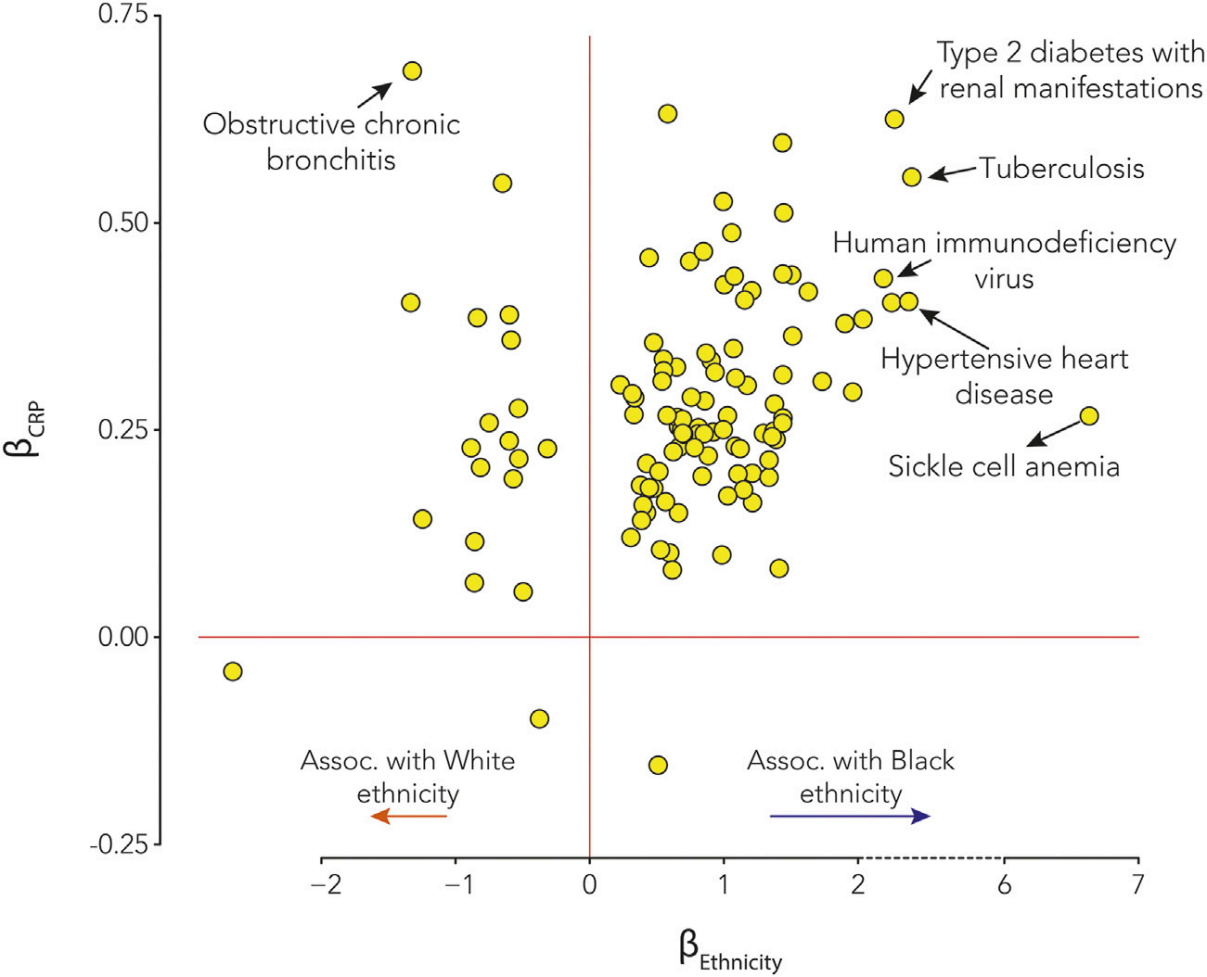
Effects of C-reactive protein (CRP) and ethnicity on disease. Effect sizes for statistically significant CRP-disease associations (β_CRP_, *y*-axis) and significant ethnicity-disease associations (β_Ethnicity_, *x*-axis) associations. β_Ethnicity_ > 0 shows diseases that are positively associated with Black ethnicity, and β_Ethnicity_ < 0 shows diseases that are positively associated with White ethnicity. Select disease examples are annotated as shown.

The relationship between CRP serum levels and ethnic health disparities was evaluated by independently modeling the effect of CRP and the effect of ethnicity on disease outcomes and comparing the results. There are 116 out of 1,537 diseases analyzed where both CRP and ethnicity showed significant associations with disease status, after correcting for multiple tests using the Bonferroni correction (Figure 4 and Supplementary Table 8). The effect size estimates for all diseases with significant CRP and ethnicity associations were evaluated to identify diseases where differences in CRP serum levels are implicated in ethnic health disparities (Figure 5). The combined effects of CRP and ethnicity on disease outcomes were quantified by summing the ranks of the individual effect sizes. The top 20 diseases ordered via descending effect size rank sums are shown in Table 3, and data for all diseases are shown in Supplementary Table 8. The top ranked diseases include examples of infectious disease (tuberculosis and HIV), metabolic diseases (type 2 diabetes and hypoglycemia), circulatory system diseases (hypertensive chronic kidney disease, hypertensive heart disease, and essential hypertension), schizophrenia, genitourinary diseases (nephrotic syndrome and chronic kidney disease), and dermatologic diseases (lupus erythematosus and sarcoidosis).

**TABLE 3:** Top 20 diseases implicated for CRP-associated ethnic health disparities.

Phenotype	Phenotype	β_CRP_	β_Ethnicity_	*p*-value_CRP_	*p*-value_Ethnicity_	Disease category
10.0	Tuberculosis	0.5553	2.4005	9.66 × 10^−19^	3.08 × 10^−26^	Infectious diseases
250.22	Type 2 diabetes with renal manifestations	0.6253	2.2715	6.93 × 10^−34^	1.26 × 10^−24^	Endocrine/metabolic
585.31	Renal dialysis	0.5122	1.4461	5.41 × 10^−50^	3.11 × 10^−15^	Genitourinary
71.0	Human immunodeficiency virus (HIV) disease	0.433	2.189	4.92 × 10^−06^	5.00 × 10^−12^	Infectious diseases
401.22	Hypertensive chronic kidney disease	0.5967	1.4365	3.25 × 10^−164^	4.28 × 10^−23^	Circulatory system
401.21	Hypertensive heart disease	0.405	2.3777	3.32 × 10^−11^	2.49 × 10^−20^	Circulatory system
295.1	Schizophrenia	0.4371	1.5061	3.09 × 10^−41^	8.25 × 10^−26^	Mental disorders
250.21	Type 2 diabetes with ketoacidosis	0.4037	2.2506	1.73 × 10^−09^	1.26 × 10^−19^	Endocrine/metabolic
580.2	Nephrotic syndrome without mention of glomerulonephritis	0.4383	1.4397	1.09 × 10^−33^	7.25 × 10^−14^	Genitourinary
697.0	Sarcoidosis	0.4169	1.6291	6.88 × 10^−36^	6.31 × 10^−27^	Dermatologic
71.1	HIV infection (symptomatic)	0.3838	2.0368	7.04 × 10^−08^	6.39 × 10^−17^	Infectious diseases
242.2	Toxic multinodular goiter	0.3785	1.9019	3.23 × 10^−06^	1.27 × 10^−08^	Endocrine/metabolic
250.42	Other abnormal glucose	0.3635	1.5125	3.76 × 10^−20^	1.17 × 10^−13^	Endocrine/metabolic
250.2	Type 2 diabetes	0.4181	1.2079	0	9.22 × 10^−195^	Endocrine/metabolic
585.3	Chronic renal failure (CKD)	0.488	1.0582	6.68 × 10^−248^	2.98 × 10^−25^	Genitourinary
695.42	Systemic lupus erythematosus	0.3087	1.7328	1.87 × 10^−13^	8.47 × 10^−23^	Dermatologic
251.1	Hypoglycemia	0.407	1.1555	1.97 × 10^−61^	1.74 × 10^−13^	Endocrine/metabolic
282.5	Sickle cell anemia	0.2726	6.7412	2.23 × 10^−08^	2.76 × 10^−224^	Hematopoietic
580.14	Chronic glomerulonephritis (NOS)	0.5258	0.9954	2.97 × 10^−67^	7.55 × 10^−06^	Genitourinary
695.41	Cutaneous lupus erythematosus	0.2959	1.9601	9.49 × 10^−06^	3.52 × 10^−14^	Dermatologic

## Discussion

### Ethnic Differences in Inflammation are Explained by Socioenvironmental Factors

C-reactive protein (CRP) is a widely used clinical marker of inflammation. Our study of the UKBB found that CRP blood serum levels differ according to participant’s self-identification as belonging to Black or White ethnic groups, and ethnicity in our cohort is highly correlated with genetic ancestry. Given these results, it could be naively expected that differences in genetic ancestry between the Black and White ethnic groups explain differences in CRP levels. However, comparisons between ethnic groups with distinct genetic ancestry profiles can be confounded by gene-environment correlations. Indeed, genetic ancestry, socioenvironmental factors, and health conditions can all co-vary among ethnic groups. We used multivariable regression and relative importance analysis in an effort to decompose the contributions of genetic ancestry, socioenvironmental factors, and health conditions to CRP ethnic disparities.

When differences in socioenvironmental exposures and health outcomes are accounted for, the associations between ethnicity, genetic ancestry, and CRP levels are almost completely attenuated. These results indicate that the environment plays a more important role than genetics in shaping ethnic disparities in inflammation for this cohort. Possible socioenvironmental factors leading to higher levels of CRP observed for Black participants could include psychosocial stress linked to racial discrimination and poverty (Sanders-Phillips et al., 2009; Williams and Mohammed 2009; Lewis et al., 2010; Beatty et al., 2014). The high relative effects of BMI and smoking on CRP suggests that aspects of diet and lifestyle associated with socioeconomic deprivation could also be linked to ethnic differences in inflammation (Feinstein 1993; Inglis et al., 2005; Govil et al., 2009).

Although we are making a semantic distinction between genetic effects, as measured by genetic ancestry, and the effects of socioenvironmental factors and health conditions, it should be noted that the socioenvironmental and health covariates modeled here are also likely be influenced by genetics. For example, BMI and smoking are both highly heritable traits. The effect of sex also likely includes a genetic component based on chromosomal differences between males and females. In other words, our approach to decomposing genetic and environmental contributions to health disparities is limited by the pervasive contributions of both genetics and the environment to human traits.

### Interaction Between Ethnicity and Sex

UKBB participant CRP blood serum levels vary by ethnicity, age, and sex. Modeling CRP levels with all of these factors reveled a highly significant interaction effect between ethnicity and sex. Black females show higher CRP levels than White females, whereas Black males have lower CRP than White males. Thus, Black females are at the highest risk of chronic inflammation, suggesting the possibility of exposure to particularly high levels of stress for this group. This finding is consistent with previous studies showing that Black women can experience worse health outcomes than Black men, White women, or White men owing to their relatively subordinate position in both ethnic and gender hierarchies (Woods-Giscombe 2010; Farmer et al., 2021). This perspective underscores the importance of an ethnic health disparities analysis framework that includes multiple, interacting demographic, genetic, and socioenvironmental factors (Bowleg 2012; Brown and Hargrove 2013; Bauer 2014; Richardson and Brown 2016).

### Inflammation and Ethnic Health Disparities

We related inflammation and ethnic health disparities by independently modeling the effect of CRP and ethnicity on disease status and then looking for diseases that showed significant associations with both factors. There were 109 out of 1,537 diseases that showed significant associations with both CRP and ethnicity, and we explored the diseases that showed the strongest effects for both. This approach uncovered a number of diseases linked to immune response and inflammation, including infectious diseases and complex, common diseases. This suggests the possibility that ethnic differences in inflammation, related to environmental exposures and psychosocial stress, could be broadly related to ethnic health disparities.

It is important to note, however, that our observational study design and statistical modelling do not allow for unambiguous causal inference regarding the relationship between CRP and disease (Davey Smith et al., 2005; Pingault et al., 2018). For infectious diseases, CRP levels are expected to be elevated after infection, which would entail a kind of reverse causality with respect to how our regression models are specified. For chronic diseases, systemic inflammation could precede disease or contribute to disease progression, but it could also reflect the presence of disease. Our models cannot distinguish between these possibilities, and it is not known whether participant CRP levels measured at recruitment precede or follow the diagnosis and course of disease. Thus, it is possible that the observed ethnic differences in CRP reflect a higher overall burden of disease for ethnic minorities in the UKBB, linked to higher levels of socioeconomic deprivation, rather than a causal risk factor for ethnic health disparities.

## Data Availability

The datasets analyzed for this study can be found on the United Kingdom Biobank website (https://www.ukbiobank.ac.uk/register-apply/).

## Footnotes

1
https://www.ukbiobank.ac.uk/learn-more-about-uk-biobank/about-us/ethics

2
https://biobank.ctsu.ox.ac.uk/crystal/field.cgi?id=21000

3
https://biobank.ctsu.ox.ac.uk/crystal/crystal/docs/serum_biochemistry.pdf

4
https://biobank.ctsu.ox.ac.uk/crystal/crystal/docs/ukb_dna_processing.pdf

## References

[B1] Abramson J. L. Weintraub W. S. Vaccarino V. (2002). Association between Pulse Pressure and C-Reactive Protein Among Apparently Healthy US Adults. Hypertension 39, 197–202. Epub 2002/02/16. 10.1161/hy0202.104270 11847183

[B2] Alley D. E. Seeman T. E. Ki Kim J. Karlamangla A. Hu P. Crimmins E. M. (2006). Socioeconomic Status and C-Reactive Protein Levels in the US Population: NHANES IV. Brain Behav. Immun. 20, 498–504. Epub 2005/12/07. 10.1016/j.bbi.2005.10.003 16330181

[B3] Banda Y. Kvale M. N. Hoffmann T. J. Hesselson S. E. Ranatunga D. Tang H. (2015). Characterizing Race/Ethnicity and Genetic Ancestry for 100,000 Subjects in the Genetic Epidemiology Research on Adult Health and Aging (GERA) Cohort. Genet. Aug. 200, 1285–1295. Epub 2015/06/21. 10.1534/genetics.115.178616 PMC4574246 26092716

[B4] Bauer G. R. (2014). Incorporating Intersectionality Theory into Population Health Research Methodology: Challenges and the Potential to advance Health Equity. Soc. Sci. Med. 110, 10–17. Epub 2014/04/08. 10.1016/j.socscimed.2014.03.022 24704889

[B5] Beatty Moody D. L. Brown C. Matthews K. A. Bromberger J. T. (2014). Everyday Discrimination Prospectively Predicts Inflammation across 7-Years in Racially Diverse Midlife Women: Study of Women's Health across the Nation. J. Soc. Issues. 70, 298–314. Epub 2014/10/25. 10.1111/josi.12061 25342861 PMC4203661

[B6] Bergström A. McCarthy S. A. Hui R. Almarri M. A. Ayub Q. Danecek P. (2020). Insights into Human Genetic Variation and Population History from 929 Diverse Genomes. Science 367, 367. Epub 2020/03/21. 10.1126/science.aay5012 PMC7115999 32193295

[B7] Black S. Kushner I. Samols D. (2004). C-reactive Protein. J. Biol. Chem. 279 (279), 48487–48490. Epub 2004/09/01. 10.1074/jbc.R400025200 15337754

[B8] Bowleg L. (2012). The Problem with the PhraseWomen and Minorities:Intersectionality-An Important Theoretical Framework for Public Health. Am. J. Public Health 102, 1267–1273. Epub 2012/05/19. 10.2105/ajph.2012.300750 22594719 PMC3477987

[B9] Brown T. H. Hargrove T. W. (2013). Multidimensional Approaches to Examining Gender and Racial/ethnic Stratification in Health. Women, Gend. Families Color. 1, 180–206. 10.5406/womgenfamcol.1.2.0180

[B10] Bycroft C. Freeman C. Petkova D. Band G. Elliott L. T. Sharp K. (2018). The UK Biobank Resource with Deep Phenotyping and Genomic Data. Nature 562, 203–209. Epub 2018/10/12. 10.1038/s41586-018-0579-z 30305743 PMC6786975

[B11] Carroll R. J. Bastarache L. Denny J. C. (2014). R PheWAS: Data Analysis and Plotting Tools for Phenome-wide Association Studies in the R Environment. Bioinformatics 30, 2375–2376. Epub 2014/04/16. 10.1093/bioinformatics/btu197 24733291 PMC4133579

[B12] Chang C. C. Chow C. C. Tellier L. C. Vattikuti S. Purcell S. M. Lee J. J. (2015). Second-generation PLINK: Rising to the challenge of Larger and Richer Datasets. GigaSci 4, 7. Epub 2015/02/28. 10.1186/s13742-015-0047-8 PMC4342193 25722852

[B13] Choudhry S. Seibold M. A. Borrell L. N. Tang H. Serebrisky D. Chapela R. (2007). Dissecting Complex Diseases in Complex Populations: Asthma in Latino Americans. Proc. Am. Thorac. Soc. 4, 226–233. Epub 2007/07/04. 10.1513/pats.200701-029aw 17607004 PMC2647623

[B14] Conley A. B. Rishishwar L. Norris E. T. Valderrama-Aguirre A. Mariño-Ramírez L. Medina-Rivas M. A. (2017). A Comparative Analysis of Genetic Ancestry and Admixture in the Colombian Populations of Chocó and Medellín. G3 Genes|Genomes|Genetics 7, 3435–3447. Epub 2017/09/01. 10.1534/g3.117.1118 28855283 PMC5633392

[B15] Danesh J. Wheeler J. G. Hirschfield G. M. Eda S. Eiriksdottir G. Rumley A. (2004). C-reactive Protein and Other Circulating Markers of Inflammation in the Prediction of Coronary Heart Disease. N. Engl. J. Med. 350, 1387–1397. Epub 2004/04/09. 10.1056/NEJMoa032804 15070788

[B16] Danner M. Kasl S. V. Abramson J. L. Vaccarino V. (2003). Association between Depression and Elevated C-Reactive Protein. Psychosomatic Med. 65, 347–356. Epub 2003/05/24. 10.1097/01.psy.0000041542.29808.01 12764206

[B17] Davey Smith G. Ebrahim S. Lewis S. Hansell A. L. Palmer L. J. Burton P. R. (2005). Genetic Epidemiology and Public Health: hope, Hype, and Future Prospects. Lancet 366, 1484–1498. Epub 2005/10/26. 10.1016/S0140-6736(05)67601-5 16243094

[B18] Dehghan A. Kardys I. de Maat M. P. M. Uitterlinden A. G. Sijbrands E. J. G. Bootsma A. H. (2007). Genetic Variation, C-Reactive Protein Levels, and Incidence of Diabetes. Diabetes 56, 872–878. Epub 2007/03/01. 10.2337/db06-0922 17327459

[B19] Elliott P. Peakman T. C. Biobank U. K. (2008). The UK Biobank Sample Handling and Storage Protocol for the Collection, Processing and Archiving of Human Blood and Urine. Int. J. Epidemiol. 37, 234–244. Epub 2008/04/03. 10.1093/ije/dym276 18381398

[B20] Fang H. Hui Q. Lynch J. Honerlaw J. Assimes T. L. Huang J. (2019). Harmonizing Genetic Ancestry and Self-Identified Race/Ethnicity in Genome-wide Association Studies. Am. J. Hum. Genet. 105, 763–772. Epub 2019/10/01. 10.1016/j.ajhg.2019.08.012 31564439 PMC6817526

[B21] Farmer H. R. Wray L. A. Haas S. A. (2021). Race, Gender, and Socioeconomic Variations in C-Reactive Protein Using the Health and Retirement Study. J. Gerontol. B Psychol. Sci. Soc. Sci. Feb. 17 (76), 583–595. Epub 2020/02/18. 10.1093/geronb/gbaa027 PMC7887729 32064519

[B22] Feinstein J. S. (1993). The Relationship between Socioeconomic Status and Health: a Review of the Literature. Milbank Q. 71, 279–322. Epub 1993/01/01. 10.2307/3350401 8510603

[B23] Ford E. S. (2002). Does Exercise Reduce Inflammation? Physical Activity and C-Reactive Protein Among U.S. Adults. Epidemiology 13, 561–568. Epub 2002/08/23. 10.1097/00001648-200209000-00012 12192226

[B24] Foster H. M. E. Celis-Morales C. A. Nicholl B. I. Petermann-Rocha F. Pell J. P. Gill J. M. R. (2018). The Effect of Socioeconomic Deprivation on the Association between an Extended Measurement of Unhealthy Lifestyle Factors and Health Outcomes: a Prospective Analysis of the UK Biobank Cohort. The Lancet Public Health 3, e576–e585. Epub 2018/11/24. 10.1016/s2468-2667(18)30200-7 30467019

[B25] Galinsky K. J. Bhatia G. Loh P.-R. Georgiev S. Mukherjee S. Patterson N. J. (2016). Fast Principal-Component Analysis Reveals Convergent Evolution of ADH1B in Europe and East Asia. Am. J. Hum. Genet. 98, 456–472. Epub 2016/03/01. 10.1016/j.ajhg.2015.12.022 26924531 PMC4827102

[B26] Genomes Project C. Auton A. Brooks L. D. Durbin R. M. Garrison E. P. Kang H. M. (2015). A Global Reference for Human Genetic Variation. Nature 526, 68–74. Epub 2015/10/04. 10.1038/nature15393 26432245 PMC4750478

[B27] Govil S. R. Weidner G. Merritt-Worden T. Ornish D. (2009). Socioeconomic Status and Improvements in Lifestyle, Coronary Risk Factors, and Quality of Life: the Multisite Cardiac Lifestyle Intervention Program. Am. J. Public Health 99, 1263–1270. Epub 2008/10/17. 10.2105/ajph.2007.132852 18923113 PMC2696652

[B28] Inglis V. Ball K. Crawford D. (2005). Why Do Women of Low Socioeconomic Status Have Poorer Dietary Behaviours Than Women of Higher Socioeconomic Status? A Qualitative Exploration. Appetite 45, 334–343. Epub 2005/09/21. 10.1016/j.appet.2005.05.003 16171900

[B29] Jordan I. K. Rishishwar L. Conley A. B. (2019). Native American Admixture Recapitulates Population-specific Migration and Settlement of the continental United States. Plos Genet. 15, e1008225. Epub 2019/09/24. 10.1371/journal.pgen.1008225 31545791 PMC6756731

[B30] Kennedy N. (2020). Forestmodel: forest Plots from Regression Models. R Package Version 06.2. https://www.ukbiobank.ac.uk/register-apply/ .

[B31] Khera A. McGuire D. K. Murphy S. A. Stanek H. G. Das S. R. Vongpatanasin W. (2005). Race and Gender Differences in C-Reactive Protein Levels. J. Am. Coll. Cardiol. 46 (46), 464–469. Epub 2005/08/02. 10.1016/j.jacc.2005.04.051 16053959

[B32] Lakoski S. G. Cushman M. Criqui M. Rundek T. Blumenthal R. S. D'Agostino R. B. Jr. (2006). Gender and C-Reactive Protein: Data from the Multiethnic Study of Atherosclerosis (MESA) Cohort. Am. Heart J. 152, 593–598. Epub 2006/08/23. 10.1016/j.ahj.2006.02.015 16923436

[B33] Lewis T. T. Aiello A. E. Leurgans S. Kelly J. Barnes L. L. (2010). Self-reported Experiences of Everyday Discrimination Are Associated with Elevated C-Reactive Protein Levels in Older African-American Adults. Brain Behav. Immun. 24, 438–443. Epub 2009/12/01. 10.1016/j.bbi.2009.11.011 19944144 PMC2826562

[B34] Mathieson I. Scally A. (2020). What Is Ancestry. Plos Genet. 16, e1008624. Epub 2020/03/10. 10.1371/journal.pgen.1008624 32150538 PMC7082057

[B35] Matthews K. A. Sowers M. F. Derby C. A. Stein E. Miracle-McMahill H. Crawford S. L. (2005). Ethnic Differences in Cardiovascular Risk Factor burden Among Middle-Aged Women: Study of Women's Health across the Nation (SWAN). Am. Heart J. 149, 1066–1073. Epub 2005/06/25. 10.1016/j.ahj.2004.08.027 15976790

[B36] Nagar S. D. Nápoles A. M. Jordan I. K. Mariño-Ramírez L. (2021). Socioeconomic Deprivation and Genetic Ancestry Interact to Modify Type 2 Diabetes Ethnic Disparities in the United Kingdom. EClinicalMedicine.37, 100960. 10.1016/j.eclinm.2021.100960 34386746 PMC8343245

[B37] Nazmi A. Victora C. G. (2007). Socioeconomic and Racial/ethnic Differentials of C-Reactive Protein Levels: a Systematic Review of Population-Based Studies. BMC Public Health 7, 212. Epub 2007/08/21. 10.1186/1471-2458-7-212 17705867 PMC2018719

[B38] Pepys M. B. Hirschfield G. M. (2003). C-reactive Protein: a Critical Update. J. Clin. Invest. 111, 1805–1812. Epub 2003/06/19. 10.1172/jci200318921 12813013 PMC161431

[B39] Pingault J.-B. O’Reilly P. F. Schoeler T. Ploubidis G. B. Rijsdijk F. Dudbridge F. (2018). Using Genetic Data to Strengthen Causal Inference in Observational Research. Nat. Rev. Genet. 19, 566–580. Epub 2018/06/07. 10.1038/s41576-018-0020-3 29872216

[B40] Richardson L. J. Brown T. H. (2016). (En)gendering Racial Disparities in Health Trajectories: A Life Course and Intersectional Analysis. SSM - Popul. Health. 2, 425–435. Epub 2017/01/24. 10.1016/j.ssmph.2016.04.011 28111630 PMC5240637

[B41] Sanders-Phillips K. Settles-Reaves B. Walker D. Brownlow J. (2009). Social Inequality and Racial Discrimination: Risk Factors for Health Disparities in Children of Color. Pediatrics 124 (Suppl. 3), S176–S186. Epub 2009/11/05. 10.1542/peds.2009-1100E 19861468

[B42] Tang H. Quertermous T. Rodriguez B. Kardia S. L. R. Zhu X. Brown A. (2005). Genetic Structure, Self-Identified Race/ethnicity, and Confounding in Case-Control Association Studies. Am. J. Hum. Genet. 76, 268–275. Epub 2004/12/31. 10.1086/427888 15625622 PMC1196372

[B43] Team R C (2013). R: A Language and Environment for Statistical Computing. Vienna: R Foundation for Statistical Computing.

[B44] Townsend P. Phillimore P. Beattie A. (1988). Health and Deprivation: Inequality and the. North: Routledge.

[B45] Welsh S. Peakman T. Sheard S. Almond R. (2017). Comparison of DNA Quantification Methodology Used in the DNA Extraction Protocol for the UK Biobank Cohort. BMC Genomics 18, 26. Epub 2017/01/07. 10.1186/s12864-016-3391-x 28056765 PMC5217214

[B46] Wickham H. (2009). Elegant Graphics for Data Analysis. Media 35, 10–1007.

[B47] Williams D. R. Mohammed S. A. (2009). Discrimination and Racial Disparities in Health: Evidence and Needed Research. J. Behav. Med. 32, 20–47. Epub 2008/11/26. 10.1007/s10865-008-9185-0 19030981 PMC2821669

[B48] Wium-Andersen M. K. Ørsted D. D. Nielsen S. F. Nordestgaard B. G. (2013). Elevated C-Reactive Protein Levels, Psychological Distress, and Depression in 73 131 Individuals. JAMA Psychiatry 70, 176–184. Epub 2012/12/26. 10.1001/2013.jamapsychiatry.102 23266538

[B49] Wong N. D. Pio J. Valencia R. Thakal G. (2001). Distribution of C-Reactive Protein and its Relation to Risk Factors and Coronary Heart Disease Risk Estimation in the National Health and Nutrition Examination Survey (NHANES) III. Prev. Cardiol. 4, 109–114. Epub 2002/02/06. 10.1111/j.1520-037x.2001.00570.x 11828186

[B50] Woods-Giscombé C. L. (2010). Superwoman Schema: African American Women's Views on Stress, Strength, and Health. Qual. Health Res. 20, 668–683. Epub 2010/02/16. 10.1177/1049732310361892 20154298 PMC3072704

[B51] Wu P. Gifford A. Meng X. Li X. Campbell H. Varley T. (2019). Mapping ICD-10 and ICD-10-CM Codes to Phecodes: Workflow Development and Initial Evaluation. JMIR Med. Inform. 7 (7), e14325. Epub 2019/09/26. 10.2196/14325 31553307 PMC6911227

[B52] Yudell M. Roberts D. DeSalle R. Tishkoff S. (2016). Taking Race Out of Human Genetics. Science 351, 564–565. Epub 2016/02/26. 10.1126/science.aac4951 26912690

[B53] Zacho J. Tybjaerg-Hansen A. Nordestgaard B. G. (2010). C-reactive Protein and All-Cause Mortality-Tthe Copenhagen City Heart Study. Eur. Heart J. 31, 1624–1632. Epub 2010/04/29. 10.1093/eurheartj/ehq103 20423919

