# Supplementary material for: Comparing Genetic and Socioenvironmental Contributions to Ethnic Differences in C-Reactive Protein
Source: Front Genet. 2021 Oct 18;12:738485. doi: 10.3389/fgene.2021.738485 (PMC8558394; doi:10.3389/fgene.2021.738485)
Supplement: Supplementary file 1 [file DataSheet1.PDF]

Supplementary Material

Comparing genetic and socioenvironmental contributions to ethnic differences in C-reactive protein

Shashwat Deepali Nagar, Andrew B. Conley, Shivam Sharma, Lavanya Rishishwar, I. King Jordan, Leonardo Mariño-Ramírez

1 Supplementary Tables

Supplementary Table 1. Global reference populations used for genetic ancestry inference of UK Biobank participants. 2

Supplementary Table 2. CRP multivariable linear regression (Model 1). 3

Supplementary Table 3. Sensitivity analysis for CRP multivariable linear regression (Model 2). 3

Supplementary Table 4. CRP multivariable logistic regression (Model 3). 3

Supplementary Table 5. CRP multivariable linear regression with interaction term (Model 4). 4

Supplementary Table 6. Likelihood ratio test for CRP multivariable linear regression models with and without ethnicity-sex interaction term. 4

Supplementary Table 7. Relative importance analysis results. 5

Supplementary Table 1. **Global reference populations used for genetic ancestry inference of UK Biobank participants.**

Geographic region	Population Name (Abbreviation)	Source[1]
African	Yoruba in Ibadan, Nigeria (YRI)	1KGP
	Esan in Nigeria (ESN)	1KGP
	Luhya in Webuye, Kenya (LWK)	1KGP
	Gambian in Western Divisions in the Gambia (GWD)	1KGP
	Mbuti in Democratic Republic of Congo (MBU)	HGDP
	Biaka in Central African Republic (BIA)	HGDP
European	Finnish in Finland (FIN)	1KGP
	British in England and Scotland (GBR)	1KGP
	Iberian Population in Spain (IBS)	1KGP
	Toscani in Italia (TSI)	1KGP
	French in France (FRE)	HGDP
	Basque in France (BAS)	HGDP
	Bergamo Italian in Bergamo, Italy (BER)	HGDP
	Tuscan in Italy (TUS)	HGDP

[1] 1KGP – 1000 Genomes Project, HGDP – Human Genome Diversity Project

Supplementary Table 2. CRP multivariable linear regression (Model 1).

Model equation, coefficient estimates, standard errors, t values, and P-values are shown.

log(CRP) = b0 + b1*Ethnicity + b2*Age + b3*Sex

Coefficient	Name	Estimate	Std. Error	t value	*P*-value
**b0**	Intercept	0.3570	0.3570	163.8244	$< 2 \times 10^{-16}$
**b1**	Ethnicity (Black)	0.0843	0.0132	6.3747	1.84×10^{-10}
**b2**	Age	0.0168	0.0002	84.3288	$< 2 \times 10^{-16}$
**b3**	Sex (Male)	-0.0654	0.0032	-20.3815	$< 2 \times 10^{-16}$

Supplementary Table 3. Sensitivity analysis for CRP multivariable linear regression (Model 2).

Individuals with CRP levels >= 10 mg/L were excluded, and the model was re-run. Model equation, coefficient estimates, standard errors, t values, and P-values are shown.

log(CRP) = b0 + b1*Ethnicity + b2*Age + b3*Sex

Coefficient	Name	Estimate	Std. Error	t value	*P*-value
b0	(Intercept)	0.2433	0.0020	122.9194	~0
b1	Ethnicity (Black)	0.0567	0.0120	4.7103	2.47×10^{-6}
b2	Age	0.0155	0.0002	85.9602	~ 0
b3	Sex (Male)	-0.0485	0.0029	-16.6613	2.63×10^{-62}

Supplementary Table 4. **CRP multivariable logistic regression (Model 3).**

Individuals with CRP levels >3 mg/L were considered to have clinically elevated levels of CRP. Model equation, coefficient estimates, standard errors, z values, and *P*-values are shown.

logit(CRP) = b0 + b1*Ethnicity + b2*Age + b3*Sex

Coefficient	Name	Estimate	Std. Error	z value	*P*-value
b0	(Intercept)	-1.1311	0.0048	-233.9251	~0
b1	Ethnicity (Black)	0.2201	0.0292	7.5440	4.56×10^{-14}
b2	Age	0.0202	0.0005	43.4897	~0
b3	Sex (Male)	-0.2560	0.0074	-34.6933	9.93×10^{-264}

Supplementary Table 5. **CRP multivariable linear regression with interaction term (Model 4).**

Model equation, coefficient estimates, standard errors, t values, and *P*-values are shown.

log(CRP) = b0 + b1*Ethnicity + b2*(Age * Sex) + b3*Sex + b4*(Ethnicity * Age) + b5*(Ethnicity * Sex) + b6*(Age * Sex) + b7*(Ethnicity * Age * Sex)

Coefficient	Name	Estimate	Std. Error	t value	P-value
b0	(Intercept)	0.3551	0.0022	162.4426	~0
b1	Ethnicity (Black)	0.1855	0.0197	9.4058	5.19×10^{-21}
b2	Age * Sex (Male)	0.0190	0.0003	68.9690	~0
b3	Sex (Male)	-0.0603	0.0032	-18.6534	1.27×10^{-77}
b4	Ethnicity (Black) * Age	-0.0050	0.0022	-2.2828	2.24×10^{-2}
b5	Ethnicity (Black) * Sex (Male)	-0.2981	0.0305	-9.7748	1.45×10^{-22}
b6	Age * Sex (Male)	-0.0045	0.0004	-11.1653	6.08×10^{-29}
b7	Ethnicity (Black) * Age * Sex (Male)	-0.0017	0.0033	-0.5292	5.97×10^{-1}

Supplementary Table 6. Likelihood ratio test for CRP multivariable linear regression models with and without ethnicity-sex interaction term.

The model equations and the likelihood test statistic values are shown below.

Model 1: log(CRP) = b0 + b1*Ethnicity + b2*Age + b3*Sex

Model 2: log(CRP) = b0 + b1*Ethnicity + b2*(Age * Sex) + b3*Sex + b4*(Ethnicity * Age) + b5*(Ethnicity * Sex) + b6*(Age * Sex) + b7*(Ethnicity * Age * Sex)

Models compared	χ^2	P-value	Δdf
**1 vs. 2**	239.91	9.7×10^{-51}	4

Supplementary Table 7. **Relative importance analysis results.**

Variable	lmg	Rank
BMI	6.16×10^{-2}	1
Age	3.09×10^{-3}	2
Smoking status	3.06×10^{-3}	3
Townsend index	1.94×10^{-3}	4
Sex	1.89×10^{-3}	5
Insomnia	9.07×10^{-4}	6
Recruitment year	1.84×10^{-4}	7
MDD	5.73×10^{-5}	8
PC1	3.23×10^{-5}	9
PC2	2.67×10^{-5}	10
Ethnicity	2.38×10^{-5}	11
